# Influential Factors Affecting Protective Coping Behaviors of Flood Disaster: A Case Study in Shenzhen, China

**DOI:** 10.3390/ijerph17165945

**Published:** 2020-08-16

**Authors:** Weiwei Cao, Yi Yang, Jing Huang, Dianchen Sun, Gaofeng Liu

**Affiliations:** 1 Institute of Management Science, Business School, Hohai University, Nanjing 211100, China; cww@hhu.edu.cn (W.C.); dcsun@hhu.edu.cn (D.S.); gaofengliu@hhu.edu.cn (G.L.); 2 Teachers College, Columbia University, New York, NY 10012, USA

**Keywords:** protective coping behaviors, flood disaster, influential factors, questionnaire survey

## Abstract

As the risk of urban flooding increases worldwide, floods seriously endanger the safety of people’s lives and property. Understanding the protective coping behaviors of the public in flood disasters is crucial to the implementation of effective flood mitigation measures and flood risk management. In this study, influential factors affecting protective coping behaviors in the face of flood disasters were identified, and the effects of these factors were discussed as well. Shenzhen City in China was selected as the study area, in which a questionnaire survey of 339 respondents was carried out in three flood-prone districts. Correlation analysis was conducted to preselect potential influential factors. Then, two linear regression models were established to identify main influential factors and to explore the interaction effects of these factors. The results indicated that age, monthly income, flood experience, trust in government and insurance willingness were main influential factors of protective coping behaviors. Trust in government had the highest positive correlation coefficient, while monthly income and age were negatively associated with protective coping behaviors. The interaction between insurance willingness and monthly income jointly affected protective coping behaviors of the public. The findings of this study could help authorities better understand the public’s intention to cope with flood and design effective risk reduction measures, not only for Shenzhen, but also for many other similar cities that facing with the same situation.

## 1. Introduction

Currently, the combined impact of various factors, such as abnormal climate and rapid urban expansion, is increasing the risk of flood disasters faced by cities [1], which seriously endangers the safety of people’s lives and property [2]. In recent years, the scale of forced evacuation due to flooding has shown a fast-growing trend worldwide. For example, in February 2020, the storm Ciara swept across Europe, causing a short period of traffic disruption, and approximately 14,000 households and companies were affected in Ireland alone. In China, the consequences of flood disasters are even more severe, due to the immature emergency management system. Flood disasters will cause numerous casualties and huge property damage without proper protective coping behaviors. Protective coping behavior is defined as the adjustment process that negative impacts of flood can be mitigated or avoided, according to the definition of the Intergovernmental Panel on Climate Change (IPCC) [3]. Understanding protective coping behaviors of the public and developing effective methods to motivate individuals and households to actively cope with flood disasters are of critical importance in flood risk management.

Studies about protective coping behaviors in flood disasters began in the 1970s. The research conducted by Huerta and Horton [4], which intended to explain the discrepancy in flood coping behaviors between old and young people, is considered as an early start to explore factors affecting people’s protective coping behaviors and has influenced subsequent studies. Reviewing relevant literatures, it can be found that studies on disaster coping behaviors mainly focused on the following aspects:

1. Studying the protective coping behaviors of different subjects. To date, the research subjects of protective coping behaviors include ordinary people [5,6], farmers [7,8], students [9,10] and tourists [11,12].
2. Identifying influential factors of protective coping behaviors. Many influential factors have been explored and analyzed, including sociodemographic factors (such as gender, age, income, education level, etc.), geographic location, previous flood experience, risk perception, trust in government, worry, knowledge of flood. Among sociodemographic factors, age, income and education level are considered to be closely related to protective coping behaviors of the public, and most research results indicate positive relationships [13,14]. Females, as a vulnerable group, are more inclined to take protective measures to deal with flood disasters compared to males, according to Cvetković’s [15] study in Serbia. In terms of geographic location, residents living close to flood risk sources are found to have higher intentions to cope with floods [16,17]. When it comes to previous flood experience, personal experience of disasters enhances the general understanding of flood risks [18], and helps victims of floods perceive more severe consequences of future flood disasters, thus leads to stronger intentions to take protective actions [19,20]. Due to the development of theoretical framework, such as protection motivation theory, the impact of risk perception on coping behaviors is also widely recognized, and this effect is usually considered to be positive [3,21]. The trust in government, is another crucial influential factor because it determines the effectiveness of the government’s risk communication and emergency management [22]. Worry is the most common emotion of the public in the face of floods, which can increase people’s intention to cope with flood risks [23,24]. With respect to the knowledge of flood, published literatures [25] believe that it is closely related to protective coping behaviors, more specifically, more knowledge leads to stronger willingness to cope. In summary, current literatures are devoted to exploring the influential factors of protective coping behaviors, but the results vary from study-to-study.
3. Studying and expanding the analytical framework of coping behaviors, such as protection motivation theory [26,27], protective action decision model [28,29], flood-risk precautionary behavior [30], etc. For example, the frame of protection motivation theory includes flood experience, barriers and socio-economic factors as additional factors to explain their influence on coping behaviors [31]. Papagiannaki et al. [30] extend current knowledge of the drivers of flood coping behaviors and predicted flood preparedness intention by proposing flood-risk precautionary behavior theory.

Current studies have explored numerous influencing factors of protective coping behaviors. Some factors are found to have direct effects, but the indirect impact of factors is often ignored. A great number of factors that indirectly affect protective coping behaviors are excluded in most studies. Moreover, studies on protective coping behaviors have a strong regionality, which produce results with local characteristics. Although many influential factors are found to have close relationships with protective coping behaviors, the influence degree of these factors differ a lot due to the variety of study areas and objects. Although structural nonstructural flood control measures are developed in China, it lacks the understanding of flood coping behaviors of the general public, thus the actual effect of the nonstructural measures is attenuated.

In order to discover the factors influencing the public’s protective intention to cope with flood disasters and how these factors influence coping behaviors in highly developed cities in China, a structured survey study was carried out in five flood-prone communities in three districts in Shenzhen. The data were collected from the proposed Likert-scale questionnaire. Given the importance of protective coping behaviors research in disaster emergency management, this study aims to identify main influential factors of protective coping behaviors and determine to which extent these factors impact the coping behaviors. This study will help government guide residents in Shenzhen to form a more proactive flood response attitude, which can be potentially applied to other cities which are similar to Shenzhen.

## 2. Materials and Methods

### 2.1. Study Area

The study was conducted in Shenzhen because it suffers from severe flood almost every year, leading to huge economic losses and widespread impacts. Meanwhile, Shenzhen is a typical large-scale and well-developed city in China, which is faced with contradictions between urban development and environmental protection. The damage caused by urban expansion to local ecological environment has increased the frequency and severity of natural disasters in Shenzhen, including flood disasters.

**Figure 1:**
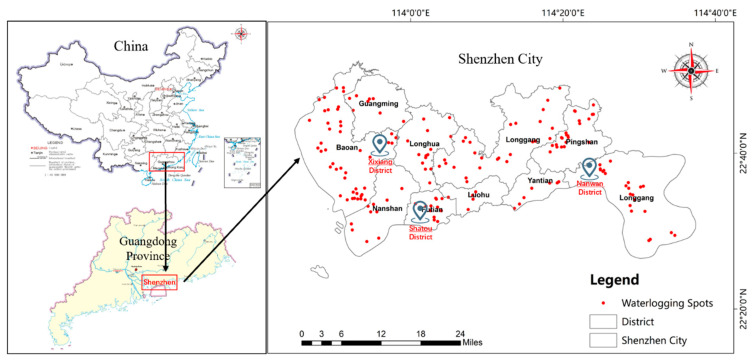
Distribution of research districts in Shenzhen, Guangdong Province, China.

Shenzhen—located in the southern region of Guangdong Province, China (as shown in Figure 1) and on the eastern shore of the Pearl River Delta—is a typical coastal developed city. It lies between 113°43′–114°38′ east longitude and 22°24′–22°52′ north latitude, with a total area of 1996.85 km^2^. The local climate type is subtropical maritime climate, with an annual average temperature of 22.4 °C and an annual average sunshine time of 2009.8 h. The annual precipitation is over 1900 mm. In addition, it suffers from 4–5 tropical cyclones (typhoons) on average each year. Shenzhen governs 9 districts including a new district and has a total resident population of 13.025 million according to the census in 2018. Shenzhen is the first Special Economic Zone in China which was established in 1980, and at present it is the core city of the Guangdong–Hong Kong–Macao Greater Bay Area (GBA). Therefore, Shenzhen is regarded as one of the national economic centers of China and the regional GDP in 2019 has reached 26,972 billion RMB, far exceeding other cities.

Shenzhen is prone to floods. Due to its coastal location and subtropical maritime climate, the frequency and the intensity of precipitation in the city is relatively high, leading to high risk of flooding. Moreover, Shenzhen is hit by typhoons frequently in the summer, exacerbating the flood risk [32]. In the history of Shenzhen, a great deal of severe flood disasters has occurred. In 2019, the occurrence of short-term heavy rainfall resulted in a sudden flood disaster in Shenzhen, causing 7 deaths and 4 missing people according to the data from Shenzhen Emergency Management Department. In 2008, a wide-range, intense and long-lasting abnormal precipitation took place, causing severe urban flooding, which affected millions of people and resulted in 8 deaths and 6 missing people. This flood event caused more than 140 waterlogging points in the city and brought about direct economic losses of about 1.2 billion RMB [33]. In 1998, Shenzhen experienced a severe once-in-a-century rainstorm, and the precipitation reached 285–292 mm in 6 h, resulting in a direct economic loss of 180 million RMB [34]. According to local statistics, it can be found that almost every ten years, a severe flood disaster appears in Shenzhen. As Cui [35] predicted using artificial intelligence algorithm, the economic losses caused by floods in Shenzhen will exceed 257 million RMB by 2020 and 309 million RMB by 2028. Therefore, there is no doubt that flood risk in Shenzhen is extremely high.

The waterlogging monitoring system for urban water accumulation in Shenzhen is complete, so it is easy to know which areas are vulnerable to waterlogging and flooding. Based on the historical data of waterlogging spots, three districts (Xixiang District, Shatou District, Nanwan District) of Shenzhen under the serious threat of flooding, were chosen to be sampling sites for the survey study. Compared with the other two districts, Nanwan District is more prone to floods, as its low-lying terrain forms waterlogging immediately after short-term heavy rainfalls. Five low-rise communities were selected near easily flooded areas because high-rise residential buildings are less affected by flood disasters.

### 2.2. Questionnaire Design

The purpose of this survey was to understand the public’s willingness to respond to flood disasters and to explore potential factors that affect the protective coping behaviors. The questionnaire consisted of 6 sections (as shown in Table 1): sociodemographic factors, risk perception, risk knowledge, risk attitudes, coping capacity and coping behaviors. There were 4 sociodemographic items, gender [15], age [36], education level [37] and monthly income [38], which mainly measured the influence of sociodemographic factors on protective coping behaviors. The second section investigated the public’s perception of flood risk [39] and local flooding likelihood [40]. The risk knowledge section, which contained flood experience [41,42] and knowledge of flood damage, aimed to assess the empirical knowledge of the respondents and to investigate their understanding of devastating floods. The next section included trust in government [30,43] and worry [23,30], in order to comprehend the respondents’ attitude toward disaster prevention of the government and their concerns about floods. The coping capacity section evaluated the coping ability of respondents, including insurance willingness [44] and familiarity of self-help measures [45]. The last section intended to measure the public’s willingness to take protective actions.

The structured questionnaire took the form of a 5-point Likert scale to facilitate the quantification of the data. The Likert scale is widely used in the field of psychology and social science to measure opinions or attitudes of respondents [46]. In order to eliminate misunderstandings about the questions, a small group of people with different education background were selected to fill out the questionnaire before the formal survey, and the items of the survey were modified to be more easily understood by respondents based on their feedback on the questions. Additionally, the reliability of the questionnaire was preliminarily tested during the pilot study, in order to ensure the overall reliability of the questionnaire.

### 2.3. Data Collection

Data were collected using a face-to-face questionnaire survey conducted from 6th July to 12th July 2019. The questionnaires were mainly distributed by graduate students who had basic knowledge of natural-disaster emergency management and the background of questionnaire design and collection. In order to ensure the number of valid questionnaires, the distribution process was led by community managers. However, the collection process adhered to the principle of voluntariness. To avoid distortion of the questionnaire data, this study did not force any respondents to fill out the questionnaire in order. Respondents had the right to refuse to participate or withdraw from the survey at any time. Moreover, a souvenir was given to each respondent after completing the questionnaire as an encouragement to their participation. The number of samples selected in each district was in accordance with the population distribution of the three target districts (the ratio of Xixiang District, Shatou District and Nanwan District is approximately 1:1:2). Eventually, a total of 400 questionnaires were distributed in the study area and 339 (84.75%) valid questionnaires were obtained excluding unqualified questionnaires.

### 2.4. Statistical Analysis Method

The process of the statistical analysis in this study can be considered as a three-stage approach. First of all, descriptive statistics were calculated to summarize the general background characteristics of the respondents (including gender, age, education level, income) and to analyze the basic distribution of the data.

Next, correlation analysis was conducted to identify whether the hypothesized influence factors had a significant impact on protective coping behaviors. The correlation coefficients of these influential factors were calculated through Equations (1) and (2), in order to examine their degrees of influence on protective coping behaviors.

(1) Cov=E[XY]−E[X]E[Y]

(2) $$R(X,Y)=\frac{Cov(X,Y)}{\sqrt{Var[X]\cdot Var[Y]}}$$

In Equation (1), E[*X*], E[*Y*] and E[*XY*] stand for the expected values of *X*, *Y* and the product of *X* and *Y*, respectively. In Equation (2), *Cov*(*X*, *Y*) denotes the covariance of *X* and *Y*, while *Var*[*X*] and *Var*[*Y*] represent the variances of *X* and *Y*, respectively.

Finally, regression analysis was conducted to determine whether there was a linear relationship between the influential factors and the protective coping behaviors. The factors of the multiple regression models were selected based on the findings of the correlation analysis. Two regression models were established and compared in order to identify main influential factors as well as interaction terms.

Both the correlation analysis and regression analysis were conducted using a significance level of 0.05, in other words, factors were considered to have an impact on protective coping behaviors when the *p*-values of these factors were less than 0.05 [38,47]. All data in this study were analyzed using SPSS statistics software (Version 22.0, SPSS, Inc., Chicago, IL, USA).

## 3. Results

### 3.1. Characteristics of Respondents

A total of 339 valid questionnaires were obtained in this study. According to the reliability test of the questionnaire data, the Cronbach’s alpha coefficient reached 0.811, which indicated good consistency of the scale [48]. Table 2 showed the sociodemographic attributes of the respondents in Shenzhen on four basic aspects: gender, age, education level and monthly income.

Among the 339 respondents, the proportion of male (50.74%) and female (49.26%) were rather close. For each district, the largest gap between male and female was less than 3%, indicating that the sample size was balanced in terms of gender. The majority (approximately 85%) of the respondents aged from 20 to 49 years old, more specifically, the proportions of people in their 20′s, 30′s and 40′s were 44.84%, 28.02% and 11.51%, respectively. With respect to education level, none of the respondents held a master’s degree or higher and high school education (36.28%) accounted for the most. In detail, most respondents in Xixiang District had middle school or below education, while most respondents held high school degree in Shatou District and bachelor’s degree in Nanwan District. Regarding monthly income, 88.79% of respondents made less than 10,000 RMB per month and only 3.54% of the respondents earned more than 20,000 RMB. The distribution of income in the three communities was basically consistent with the overall population. On the whole, the respondents in the three districts were representative of the general population.

### 3.2. Protective Coping Behaviors in Three Districts

Among the 339 samples, 89 respondents (26.25%) were from Xixiang District, 72 (21.24%) were from Shatou District and 178 (52.51%) were from Nanwan District, which was consistent with the population distribution of the three districts (the population of each district was approximately 66,000, 63,000 and 122,000, respectively, about 1:1:2). Table 3 showed the descriptive statistics of the respondents’ intentions to take protective coping behaviors in three areas.

As shown in Table 3, the overall mean score of the 339 respondents’ intentions to take protective coping behaviors in Shenzhen was 3.76 (SD = 1.225) on a scale of 1–5. This means that respondents in Shenzhen had a slightly higher than medium level of coping behavior intention during flood disasters, which further indicated a greater risk of potential flood loss in Shenzhen. Nanwan District had the highest level of coping behavior intention, with mean of 3.85 (SD = 1.176), exceeding the grand mean, which could be explained by being one of the most severe flood-prone areas in Shenzhen. On the contrary, the levels of coping behavior intention of Xixiang District and Shatou District were lower than average, with mean of 3.71 (SD = 1.168) and 3.64 (SD = 1.359), respectively. However, it was found that the *p*-values of the pairwise comparisons were all greater than 0.05 (Xixiang District and Shatou District: t(336) = −0.350, *p* = 0.727; Xixiang District and Nanwan District: t(336) = −1.307, *p* = 0.192; Shatou District and Nanwan District: t(336) = −0.818, *p* = 0.414), which indicated that the differences of the levels of protective coping behaviors among the three districts was insignificant.

### 3.3. Correlation between Factors

The purpose of the correlation analysis between each influential factors and protective coping behaviors was twofold: (a) to verify whether the influential factors in Table 1 were associated with protective coping behaviors; (b) to find out how these factors were correlated with protective coping behaviors.

As shown in Table 4, gender, perception of local flooding likelihood and monthly income proved to be unrelated to protective coping behaviors, because the *p*-values of these factors were all greater than 0.05 and the correlation coefficients were all less than 0.1. Correlation analysis failed to provide evidence for the impact of the gender and perception of local flooding likelihood on protective coping behaviors of the public. Moreover, monthly income was also considered to be independent from protective coping behaviors through the correlation analysis. This result was similar to the findings of Meyer et al. [38], which discovered that there was no significant correlation between income and evacuation intention.

Age, education level, flood risk perception, flood experience, knowledge of flood damage, trust in government, worry, insurance willingness, familiarity of self-help measures passed the correlation test, which indicated that changes in these factors will affect protective coping behaviors when people are faced with flood disasters. Among these factors, only age was negatively correlated with protective coping behaviors—that was, the willingness to cope protectively diminished as people got older. Except for age, all the other factors were positively correlated with protective coping behaviors.

**Figure 2:**
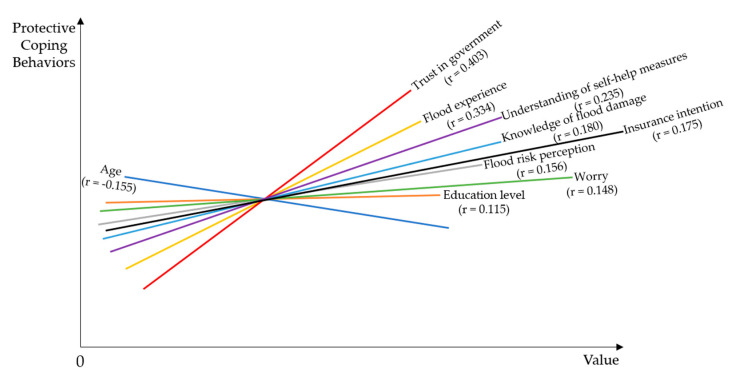
Schematic diagram of correlation between factors.

Figure 2 intuitively showed the correlations between each factor and protective coping behaviors, from which we could tell the ranking of the degree of correlation. In terms of the correlation degree, the five factors that had the greatest influences on protective coping behaviors were: trust in government (r = 0.403), flood experience (r = 0.334), familiarity of self-help measures (r = 0.235), knowledge of flood damage (r = 0.180), insurance willingness (r = 0.175). The correlation coefficients of trust in government and flood experience were greater than 0.3, indicating that they were the key factors affecting coping behaviors of the public. Additionally, the impact of other factors on protective coping behaviors was not neglectable. The correlations with familiarity of self-help measures, knowledge of flood damage, insurance willingness, flood risk perception, worry and education level were 0.235, 0.180, 0.175, 0.156, 0.148 and 0.115, respectively. A weak correlation between flood risk perception and protective coping behaviors was found in this study.

### 3.4. Influential Factors of Protective Coping Behaviors

(1) Linear regression model

Regression analysis was carried out to explore the linear relationships between the influential factors and protective coping behaviors of the residents in Shenzhen. Initially, a multiple regression model which contained 9 influential factors was established with a significance level of 0.05.

Factors that passed the correlation test (age, education level, flood risk perception, flood experience, knowledge of flood damage, trust in government, worry, insurance willingness and familiarity of self-help measures) were considered to be closely related to protective coping behaviors. Therefore, all these factors were included in the initial multiple linear regression model. The results showed that this model was significant (*F*(9, 329) = 11.459, *p* = 0.000) to predict protective coping behaviors. The adjusted R-square was 0.218, which means 21.8% variation in the dependent variable (protective coping behaviors) could be explained by the regression.

As shown in Table 5, age (*p* = 0.010), flood experience (*p* = 0.002) and trust in government (*p* = 0.000) were regarded as main influential factors of protective coping behaviors in this model, because the significance level of these three factors were all less than 0.05. However, the other variables (education, flood risk perception, knowledge of flood damage, worry, insurance willingness and familiarity of self-help measures) were insignificant in predicting protective coping behaviors. Nevertheless, the influence of these factors on protective coping behaviors was still worth discussing.

The regression coefficient (B) measured the linear effect level of independent variables on coping behaviors. The regression coefficients of trust in government, 0.330, was the highest in this model, followed by flood experience, which was 0.172. The regression coefficient of age was negative (B = −0.162), which was consistent with the results from the correlation analysis. Among the insignificant factors, the regression coefficients of worry (B = 0.084) and familiarity of self-help measures (B = 0.093) were nonignorable.

To summarize, through the multiple linear regression model, three main influential factors of protective coping behaviors, age, flood experience and trust in government, were identified. Therefore, when studying coping behaviors of the public, these three factors should be taken into account. Even though the other factors were found to be insignificant in this linear regression, it did not mean that they had no effect on coping behaviors. In the next section, the interaction effects of these variables were studied, instead of simply excluding them from the linear model.

(2) Linear regression model with interaction

Although the linear regression model in Section 3.3 only identified three major influential factors of protective coping behaviors, this model ignored the interaction effects of these factors. It did not necessarily suggest that insignificant variables should be eliminated from the linear model, and they might potentially interact with other variables to jointly influence coping behaviors. Therefore, this study attempted to explore all possible linear models with interactions. An interaction term was the product of two different variables, and only the interaction between monthly income and insurance willingness were presented.

Compared to the initial linear regression model above, the new model considering interaction effect improved the goodness of fit (adjusted R-square = 0.2261), indicating 0.81% more of the variability in the protective coping behaviors could be explained by including the interaction between monthly income and insurance willingness in the model. As shown in Table 6, the interaction between monthly income and insurance willingness was significant (*p* = 0.027). This indicated that monthly income and insurance willingness jointly affected the protective coping behaviors, even though neither of them was significant in the previous model. This finding agreed with the significant correlation coefficient between monthly income and insurance willingness (as shown in Table 4) which suggested that these two factors were closely associated.

**Figure 3:**
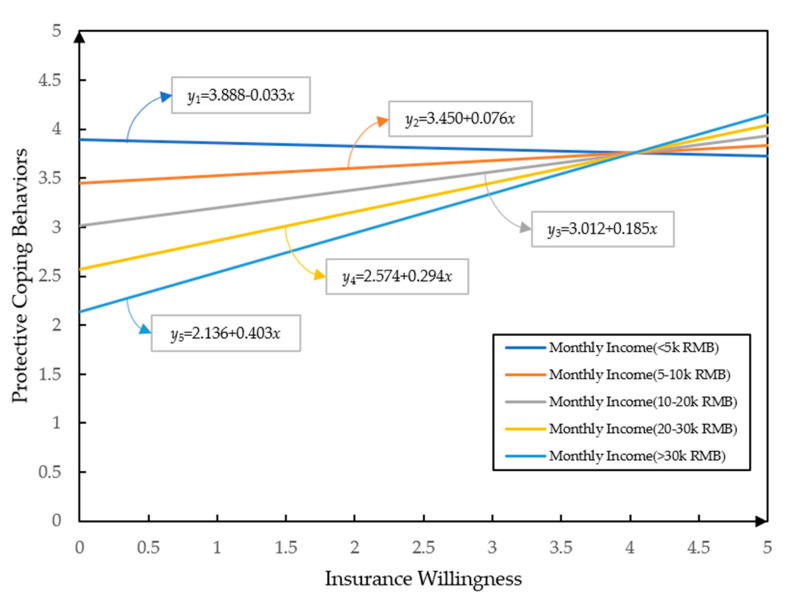
The interaction effect of monthly income and insurance willingness. Note: *y* represents protective coping behaviors, *x* represents insurance willingness.

In order to further explore the interaction effect between monthly income and insurance willingness, other factors in the model, age, trust in government and flood experience, were fixed as constant. The values of age (mode = 2), trust in government (mode = 3), flood experience (mode = 5) were set to be their modes. As demonstrated in Figure 3, when the monthly income of the respondents was less than 5000 RMB, their intentions to cope protectively decreased as their insurance willingness increased. On the contrary, when monthly income was more than 5000 RMB, there was a positive relationship between protective coping behaviors and insurance willingness. The higher the income level was, the greater the positive effect of insurance intention on protective coping behaviors. In other words, the willingness to take coping behaviors of high-income individuals was stronger when they had higher insurance willingness, while that of low-income people was stronger when they had lower insurance willingness.

## 4. Discussion

This study aimed to explore which factors have significant impact on protective coping behaviors. Age, education level, flood risk perception, flood experience, knowledge of flood damage, trust in government, worry, insurance willingness, and familiarity of self-help measures were found to be correlated with protective coping behaviors of the residents in Shenzhen. In addition, we also found out that main influential factors, such as age, trust in government and flood experience, had a strong linear relationship with coping behaviors, which was consistent with most findings from studies in other countries, for instance, Thailand, New Zealand, etc. [49,50,51].

More important, factors that were not highly correlated with protective coping behaviors might also affect coping behaviors. For example, monthly income and insurance willingness were found to be insignificant in the initial regression model, but after including the interaction term of these two factors in the second regression model, their impact on protective coping behaviors became significant. This could be explained by similar studies, which had found that monthly income and insurance willingness were regarded as nonnegligible factors of coping behaviors [47,52]. Therefore, when screening factors, correlation analysis or simple linear analysis were not sufficient to determine the potential influence of factors. In other words, the factors could not be excluded only based on the results of these analyses. The interaction effects of factors needed to be further taken into account.

The structural equation model (SEM) is a common tool in other studies to explore the complicated relationships among factors. For example, a similar study conducted by Huang et al. [53], established a SEM to explore the relationship between influential factors and protective coping behaviors. Huang’ study found that protective coping behaviors of the public were indirectly affected by sociodemographic factors like age and monthly income, which suggested that sociodemographic factors might interact with other influential factors on protective coping behaviors. This study proposed another approach to explore the interaction effects between variables using linear regression, and similar results were discovered. Monthly income—as one of the typical sociodemographic factors—jointly affected the protective coping behaviors with insurance willingness. On top of this, the potential interaction of the effects of other factors on protective coping behaviors could be further investigated using the proposed method.

In this study, only three flood-prone districts in Shenzhen were selected. Therefore, these findings only represented the protective coping behaviors of people under serious threats of flooding. In the future, more samples will be selected from the overall population in order to reach more general conclusions. The questionnaire survey was carried out anonymously to ensure the objectivity of the results. However, some illiterate participants finished the questionnaires with the assistance of our volunteers, thus they might feel uncomfortable sharing their true opinions, especially government-related questions, leading to biased ratings of these items. Furthermore, because there is no standardized questionnaire of protective coping behaviors, this study may have omissions in the selection of influencing factors. A standardized questionnaire should to be developed and validated in future studies. Nonetheless, findings of this research could help decision-makers in the government to formulate effective communication strategies and flood risk reduction policies. In the future, we will include more samples and explore the coping behaviors of the public in depth from a social point of view.

## 5. Conclusions

In summary, five flood-prone communities from three districts in Shenzhen were investigated and more than 300 valid questionnaires were analyzed. First, the overall level of protective coping behaviors of respondents in the three districts was analyzed. Then, the correlation analysis and regression analysis were conducted to determine which factors influence protective coping behaviors of the public in Shenzhen. The key findings of this study are:

1. Respondents’ protective coping behaviors in Shenzhen were above the medium level with overall mean of 3.76 (on a scale from 1 to 5). More specifically, Nanwan District had the highest level of protective coping behaviors, with mean of 3.85, and Xixiang District had the lowest level of protective coping behaviors, with mean of 3.64;
2. Age, flood experience, trust in government, monthly income and insurance willingness were found to be closely associated with protective coping behaviors in Shenzhen. Trust in government (B = 0.360) had the greatest positive impact, followed by flood experience (B = 0.205). Age (B = −0.157) and monthly income (B = −0.438) were negatively associated with protective coping behaviors;
3. Monthly income and insurance intention had insignificant direct effect on protective coping behaviors. However, the interaction effect of these two factors on protective coping behaviors was significant, which indicated that insurance willingness and monthly income (B = 0.109) jointly affect the protective coping behaviors.

This study concluded that age, flood experience, trust in government, monthly income and insurance willingness were the main influential factors of protective coping behaviors of residents in Shenzhen. We conducted a 3-step approach to explore the interaction between variables using linear model and explored the interaction of monthly income and insurance willingness. These findings can help authorities better understand the public’s intention to cope with flood disasters and design effective risk reduction measures, not only for Shenzhen, but also for many similar cities in China faced with the same situation and dilemma.

## Figures and Tables

**Table 1:** Definition of measurement and influential factors of protective coping behaviors.

Section	Variables	Detail
Sociodemographic factors	Gender	The basic social background characteristics of respondents
	Age	
	Education level	
	Monthly income	
Risk perception	Flood risk perception	Respondents’ perception of flood risk
	Perception of local flooding likelihood	
Risk knowledge	Flood experience	Respondents’ empirical knowledge and understanding of the destructiveness of flood
	Knowledge of flood damage	
Risk attitudes	Trust in government	Respondents’ trust in the government’s disaster prevention and their concern about floods
	Worry	
Coping capacity	Insurance willingness	Evaluation of respondents’ coping ability
	Familiarity of self-help measures	
Coping behaviors	Protective coping behaviors	Respondents’ willingness to take protective actions

**Table 2:** Sociodemographic attributes of respondents.

Variable	Shenzhen (Total)	Xixiang District	Shatou District	Nanwan District
Gender *n* (%)				
Male	172 (50.74%)	44 (49.44%)	37 (51.39%)	91 (51.12%)
Female	167 (49.26%)	45 (50.56%)	35 (48.51%)	87 (48.88%)
Age *n* (%)				
<20 years old	30 (8.84%)	6 (6.74%)	15 (20.83%)	9 (5.06%)
20–29 years old	152 (44.84%)	28 (31.46%)	33 (45.83%)	91 (51.12%)
30–39 years old	95 (28.02%)	30 (33.71%)	13 (18.06%)	52 (29.21%)
40–49 years old	39 (11.51%)	16 (17.98%)	7 (9.72%)	16 (8.99%)
≥50 years old	23 (6.79%)	9 (10.11%)	4 (5.56%)	9 (5.62%)
Education level *n* (%)				
Middle school or below	102 (30.09%)	40 (44.95%)	21 (29.17%)	49 (27.53%)
High school	123 (36.28%)	34 (38.20%)	32 (44.44%)	51 (28.65%)
Bachelor’s or higher	114 (33.63%)	15 (16.85%)	19 (26.39%)	78 (43.82%)
Monthly income *n* (%)				
<¥5000	191 (56.34%)	51 (57.30%)	31 (43.06%)	109 (61.24%)
¥5000–9999	110 (32.45%)	20 (22.47%)	32 (44.44%)	58 (32.58%)
¥10,000–19,999	26 (7.67%)	16 (17.98%)	4 (5.56%)	6 (3.37%)
¥20,000–29,999	7 (2.06%)	2 (2.25%)	3 (4.17%)	2 (1.12%)
≥¥30,000	5 (1.48%)	0 (0.00%)	2 (2.77%)	3 (1.69%)

**Table 3:** Comparison of protective coping behavior intention between different districts.

District	Mean	N	Std. Deviation	Difference from Mean
Xixiang	3.640	89	1.359	−0.110
Shatou	3.710	72	1.168	−0.050
Nanwan	3.850	178	1.176	0.090
Total	3.760	339	1.225	-

**Table 4:** Results of correlation analysis between influential factors and protective coping behaviors.

	PCB	Gen	Age	EL	MI	FRP	PLFL	FE	KFD	TG	Wor	IW	FSM
**PCB**	1												
**Gen**	0.055	1											
**Age**	−0.155 **	−0.070	1										
**EL**	0.115 **	−0.007	−0.299 **	1									
**MI**	−0.015	−0.050	−0.031	0.122 *	1								
**FRP**	0.156 **	−0.127 *	0.009	0.227 **	0.072	1							
**PLFL**	−0.069	0.063	0.070	0.069	−0.029	0.145 **	1						
**FE**	0.334 **	0.045	−0.119 *	0.158 **	0.101	0.305 **	0.071	1					
**KFD**	0.180 **	−0.065	0.036	0.121 *	0.068	0.386 **	0.074	0.384 **	1				
**TG**	0.403 **	−0.029	−0.054	0.045	0.038	0.195 **	−0.072	0.321 **	0.259 **	1			
**Wor**	0.148 **	0.117 *	0.117 *	0.038	0.089	0.043	0.131 *	0.155 **	0.160 **	0.126 *	1		
**IW**	0.175 **	0.027	−0.215 **	0.329 **	0.143 **	0.202 **	−0.006	0.247 **	0.177 **	0.198 **	0.163 **	1	
**FSM**	0.235 **	−0.053	0.060	0.068	0.034	0.465 **	0.146 **	0.371 **	0.545 **	0.293 **	0.129 *	0.094	1

Note: PCB—protective coping behaviors; Gen—gender; EL—education level; MI—monthly income; FRP—flood risk perception; PLFL—perception of local flooding likelihood; FE—flood experience; KFD—knowledge of flood damage; TG—trust in government; Wor—worry; IW—insurance willingness; FSM—familiarity of self-help measures. ** represents that the correlation is significant at the 0.01 level (2-tailed); * represents that the correlation is significant at the 0.05 level (2-tailed).

**Table 5:** Coefficients of the linear regression model.

Regression Model	B	Std. Error	t	*p*-Value
Constant	1.569	0.382	4.108	0.000 **
Age	−0.162	0.063	−2.583	0.010 **
Education level	0.031	0.081	0.386	0.700
Flood risk perception	0.003	0.062	0.044	0.965
Flood experience	0.172	0.055	3.123	0.002 **
Knowledge of flood damage	−0.034	0.067	−0.515	0.607
Trust in government	0.330	0.057	5.788	0.000 **
Worry	0.084	0.048	1.742	0.082
Insurance willingness	0.014	0.046	0.310	0.756
Familiarity of self-help measures	0.093	0.065	1.433	0.153

Note: ** represents that the correlation is significant at the 0.01 level.

**Table 6:** Coefficients of Model with interactive variables.

Model with Interactive Variables	B	Std. Error	t	*p*-Value
Constant	2.535	0.417	6.081	0.000 **
Age	−0.157	0.059	−2.653	0.008 **
Trust in government	0.360	0.056	6.499	0.000 **
Flood experience	0.205	0.051	4.044	0.000 **
Monthly income	−0.438	0.174	−2.512	0.012 *
Insurance willingness	−0.142	0.091	−1.562	0.119
Monthly income * insurance willingness	0.109	0.049	2.221	0.027 *

Note: ** represents that the correlation is significant at the 0.01 level; * represents that the correlation is significant at the 0.05 level.
